# Validation of a Prediction Model for Intraoperative Hypothermia in Patients Receiving General Anesthesia

**DOI:** 10.1155/2022/6806225

**Published:** 2022-09-17

**Authors:** Ziyi Dai, Yuelun Zhang, Jie Yi, Yuguang Huang

**Affiliations:** ^1^Department of Anesthesiology, Peking Union Medical College Hospital, Chinese Academy of Medical Sciences and Peking Union Medical College, Beijing 100730, China; ^2^Medical Research Center, Peking Union Medical College Hospital, Chinese Academy of Medical Sciences and Peking Union Medical College, Beijing 100730, China

## Abstract

**Objectives:**

There have been no fully validated tools for the rapid identification of surgical patients at risk of intraoperative hypothermia. The objective of this study was to validate the performance of a previously established prediction model in estimating the risk of intraoperative hypothermia in a prospective cohort.

**Methods:**

In this observational study, consecutive adults scheduled for elective surgery under general anesthesia were enrolled prospectively at a tertiary hospital between September 4, 2020, and December 28, 2020. An intraoperative hypothermia risk score was calculated by a mobile application of the prediction model. A wireless axillary thermometer was used to continuously measure perioperative core temperature as the reference standard. The discrimination and calibration of the model were assessed, using the area under the receiver operating characteristic curve (AUC), Hosmer–Lemeshow goodness-of-fit test, and Brier score.

**Results:**

Among 227 participants, 99 (43.6%) developed intraoperative hypothermia, and 10 (4.6%) received intraoperative active warming with forced-air warming. The model had an AUC of 0.700 (95% confidence interval [CI], 0.632–0.768) in the overall cohort with adequate calibration (Hosmer–Lemeshow *χ*^2^ = 13.8, *P*=0.087; Brier score = 0.33 [95% CI, 0.29–0.37]). We categorized the risk scores into low-risk, moderate-risk, and high-risk groups, in which the incidence of intraoperative hypothermia was 23.0% (95% CI, 12.4–33.5), 43.4% (95% CI, 33.7–53.2), and 62.7% (95% CI, 51.5–74.3), respectively (*P* for trend <0.001).

**Conclusions:**

The intraoperative hypothermia prediction model demonstrated possibly helpful discrimination and adequate calibration in our prospective validation. These findings suggest that the risk screening model could facilitate future perioperative temperature management.

## 1. Introduction

Inadvertent intraoperative hypothermia, defined as a core temperature <36.0°C at any point during the operation [1], is present in 4% to 90% of surgical patients [2, 3]. Hypothermia can lead to numerous adverse outcomes, including postoperative infection [4, 5], cardiovascular events [5, 6], increased blood loss and transfusion requirement [7], and altered pharmacodynamics [8], with substantial costs [5, 9]. Professional societies, such as the National Institute for Health and Care Excellence (NICE) and the American Society of PeriAnesthesia Nurses (ASPAN), have submitted some clinical guidelines [1, 10] for the management of perioperative hypothermia and recommended forced-air warming as the most effective active warming [3, 10].

Nevertheless, temperature monitoring and active warming have not been part of routine perioperative care for all surgical patients in many countries yet, and intraoperative hypothermia remains a common problem worldwide [11–13]. A study [11] in the United States reported that intraoperative temperature measurement was discontinued >30 minutes before anesthesia ending in up to 59.5% of cases. In China, 44.3% of patients undergoing elective operations with general anesthesia developed intraoperative hypothermia, whereas intraoperative active warming was applied to only 14.2% of patients [12]. Moreover, routine use of active body warming in addition to other warming care provides no added benefit in preventing intraoperative hypothermia in short procedures [14, 15]. Preoperative screening for persons at “high risk” of intraoperative hypothermia may help identify patients with more need of warming resources and temperature monitoring, in an effort to guide perioperative temperature management, enhance patient safety, and improve clinical outcomes; however, there are no such prospectively validated predictive tools that we know of.

A multicenter, cross-sectional study [12] in China of 3132 patients receiving general anesthesia monitored core temperature perioperatively. We identified the risk factors of intraoperative hypothermia [12] and established a prediction equation, requiring several clinical variables related to patients' basic condition, anesthesia management, surgery categories, and ambient temperature, to estimate the absolute risk of hypothermia [16]. A mobile application (APP), “Intraoperative Hypothermia Predictor APP” (Dacheng Medical, Jiangmen, China; http://www.iobmedical.com.cn), was then developed based on a modified version of the prediction model. Through a retrospective validation [17], using Beijing regional survey data, the risk-calculation model performed well in predicting intraoperative hypothermia [16].

The aim of this study was to validate the prediction model in patients undergoing general anesthesia using a prospective cohort before further implementation of the model. Our primary hypothesis was that the prediction model has helpful discrimination and adequate calibration for clinical use.

## 2. Materials and Methods

Ethical approval for the study was obtained from the Institutional Review Board (IRB) of Peking Union Medical College Hospital (JS-1700), and all participants provided written informed consent. The study adhered to the Standards for Reporting Diagnostic Accuracy Studies (STARD) reporting guidelines [18].

### 2.1. Study Population

This single-center, prospective, observational cohort study was conducted in Peking Union Medical College Hospital, a Class A tertiary general hospital with an annual volume of approximately 54,000 operations in Beijing, China. Participants aged 18 years or older, with scheduled operations in preselected operating rooms, were enrolled consecutively by one anesthesiologist (DZY) in the research team from September 4, 2020, through December 28, 2020. Adults undergoing elective operations that were expected to last more than 40 minutes with general anesthesia were eligible for inclusion. We excluded patients who had central hyperthermia (e.g., cerebrovascular disease, traumatic brain injury, cerebral operation, epilepsy, or acute hydrocephalus), had impaired thermoregulation (e.g., neuroleptic malignant syndrome or malignant hyperthermia), had known hyperthyroidism (or hypothyroidism) with current thyroid dysfunction, had infectious fever, had core temperature ≥38.5°C attributable to other causes within 3 days before surgery, were unsuited for infrared tympanic thermometry or axillary temperature monitoring, were scheduled for operation with induced hypothermia (e.g. cardiopulmonary bypass), or were unwilling to give signed consent [12, 16].

### 2.2. Procedures

To provide reliable results, designated staff anesthesiologists and operating room nurses were trained for temperature measurement, risk score calculation, and data collection (details are given as follows).

Anesthetics and perioperative temperature management were chosen at the discretion of the anesthesiologists. General anesthesia was mostly induced with 2–2.5 mg/kg propofol, 2–4 *μ*g/kg fentanyl, and 0.8–1 mg/kg rocuronium and maintained with sevoflurane at a dose of 1.5–2 vol % mixed with O_2_/N_2_O (50%/50%). Room temperature intravenous fluid, warm blood products, and warm irrigation fluid were administered as routine practice. All participants were covered with cotton blankets, sheets, or surgical drapes per usual care perioperatively. The participants were actively warmed using active heating devices (Bair Hugger Warming Unit Model 775; 3M, St. Paul, MN) and forced-air warming blankets (Bair Hugger full access underbody blanket model 635; 3M, St. Paul, MN) placed under them, as the individual decision of the anesthesiologists. Ambient temperature was set according to surgical preference and was measured with an electronic probe (Fluke 971 Temperature Humidity Meter; Fluke Corporation, Everett, WA), which was placed close to the participants and away from any heat-producing equipment.

At admission to the preoperative holding area, a noninvasive wireless thermometer (iThermonitor WT701; Raiing Medical, Boston, MA) was inserted and patched in a shaved axilla. The thermometer was paired to a module that continuously recorded axillary temperature, and temperature data were uploaded to a terminal server for unified storage. Temperature readings were displayed on the electrocardiogram monitor (M8007 A Patient Monitor; Philips Medizin Systeme Boeblingen GmbH, Boeblingen, Germany) wirelessly connected to the thermometer. The iThermonitor axillary temperature was tested as a sufficiently accurate and precise core temperature measurement in a previous study by Pei et al. [19], which was also suggested as near-core thermometry by Sessler [20]. On arrival in the OR, patients' axillary temperatures were immediately measured at baseline before anesthesia induction. In some cases, according to anesthesiologists' choice, a calibrated and validated infrared tympanic thermometer [20] (Thermo Scan PRO-4000; Braun GmbH, Kronberg, Germany) with disposable covers was applied to obtain baseline core temperature for calculation of the risk score. Core temperature was documented by anesthesiologists every 5 or 15 minutes throughout the operation. Axillary thermometers were removed before participants were discharged from the postanesthesia care unit (PACU) or transferred to the intensive care unit (ICU).

Hypothermia risk assessment was carried out using the Intraoperative Hypothermia Predictor APP (Supplemental Digital Content, eFigure 1) by the anesthesiologists just before surgery or by an investigator who was blinded to all intraoperative and postoperative results, including the body temperature data. To calculate the risk score, 13 clinical variables (age, sex, height, weight, American Society of Anesthesiologists [ASA] physical status, the magnitude of surgery, the mode of anesthesia, the estimated volume of intraoperative intravenous fluid, the estimated volume of irrigation fluid, the estimated duration of anesthesia, minimally invasive surgery, preoperative baseline core temperature, and OR ambient temperature) were inputted into the Predictor APP. The risk score from the APP ranges from 0 to 100, with 100 indicating the highest probability of intraoperative hypothermia and 0 indicating the least [16].

### 2.3. Statistical Analysis

Intraoperative hypothermia was defined as a core temperature <36.0°C at any time in the perioperative period, consistent with the previous studies [12, 17] and the NICE guideline [1]. Core temperature data were occasionally missing completely at random [21] due to the axillary thermometer falling off or other accidental reasons, which were independent of other clinical characteristics. Participants whose temperature data were missing were excluded from the analysis because the variance of the ambient temperature was relatively small in the same season at our institution, mean imputation [21] was used for missing ambient OR temperatures to calculate risk scores.

The risk scores were transformed using the logit function, which was normally distributed in the whole cohort after logit transformation (Supplemental Digital Content, eFigure 2), using the following formula:

(1) $$\begin{array}{c} \text{Log}it\;S=\log\frac{\text{score}\%}{1-\text{score}\%}. \end{array}$$

The 33.3% and 66.7% quantiles of Logit S were 0.917 and 2.066, respectively. We then converted the two thresholds back to the original risk scores, that is, 71.5 and 88.8. Considering convenience in clinical practice, we simplified the lower and upper cutoffs to 70 and 90, and participants were classified into 3 groups according to their intraoperative hypothermia risk scores: low risk, 70 or less; moderate risk, greater than 70 to less than 90; high risk, 90 or greater.

We used descriptive statistics to explore the distribution of participant characteristics, intraoperative temperature, and warming modalities. Discrimination [22] of the model was evaluated with ROC analyses for the overall cohort to assess the ability to predict intraoperative hypothermia. The area under the ROC curve (AUC) was calculated with a 95% confidence interval (CI) by use of the Hanley–McNeil nonparametric method [23]. Based on Users' Guides to the Medical Literature from *JAMA* [22], as is generally accepted, an AUC between 0.60 and 0.75 indicates possibly helpful discrimination, and an AUC of more than 0.75 is considered clearly useful. Accuracy metrics, including sensitivities, specificities, positive predictive values (PPVs), negative predictive values (NPVs), positive likelihood ratios (LR + *s*), and negative likelihood ratios (LRs) at two score cutoffs with corresponding 95% CIs, were also computed. Intraoperative hypothermia incidence, relative risks (RRs), and likelihood ratios (LRs) [24] were calculated within the 3 risk groups, and a test for linear trend was performed using logistic regression. The agreement for intraoperative hypothermia risk between observations and estimations by decile was assessed with the Hosmer–Lemeshow goodness-of-fit test and visually represented with a calibration plot [22, 25]. A two-sided *P* value of less than 0.05 suggests poor calibration. Furthermore, we calculated the Brier score to demonstrate the overall model performance, with 0 for a perfect model, 1 being the worst [25], and the 95% CI was estimated by bootstrapping with 1,000 resamples. Sensitivity analyses for warming status, from which we excluded participants with intraoperative active warming, were performed. We also conducted analyses to evaluate thermometer sensitivity by applying the model to only the participants with baseline temperature acquired from the axilla. Two-sided *P* < 0.05 was considered statistically significant. Statistical analyses were performed with SPSS statistical software version 24.0 (IBM Corporation).

Calculation of the sample size was based on an estimated intraoperative hypothermia incidence of 39.9%, as has been reported [16, 17]. With a two-tailed test at a significance level of 5%, a required sample size of 211 participants would provide a power of 90% to detect a significant increase of AUC from 0.650 to 0.771 (using PASS version 11.0.7). An AUC of 0.650 has been determined as having no clinical relevance. The estimate of 0.771 was derived from a previous validation of the prediction equation in a retrospective cohort [16].

### 2.4. Data Collection

Data were collected perioperatively via case report forms completed by staff managing the participants. Demographic characteristics such as age, sex, height, weight, body mass index (BMI), and ASA physical status were collected. BMI was calculated as weight in kilograms divided by the square of height in meters. We also prospectively recorded details of anesthesia and surgery: preoperative diagnosis, surgical procedure, mode of anesthesia, duration of surgery, duration of anesthesia, intraoperative intravenous fluid, irrigation fluid, blood transfusion, blood loss, and ambient temperature in the preoperative area, OR, and PACU. Other temperature statistics collected were as follows: preoperative baseline core temperature, the type of thermometer used at baseline (iThermonitor, others), intermittent intraoperative core temperature, and risk score. Continuous core temperature data during the observation period were accessed through the terminal server. As reported [12, 17], the magnitude of surgery was classified as minor, intermediate, major, and major-plus. The use of warming modalities, classified as passive warming (cotton blankets, sheets, and surgical drapes) and active warming (fluid warmers and forced-air warming) [17], was recorded.

## 3. Results

**Figure 1:**
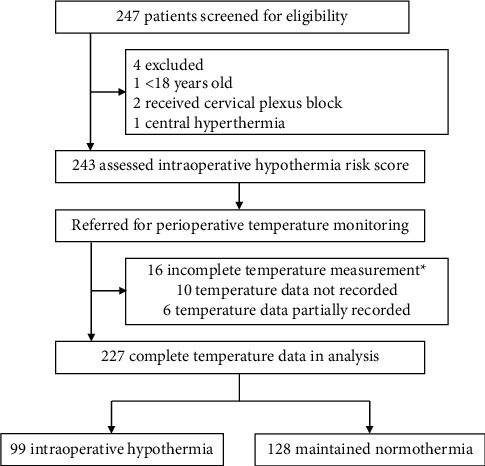
Flow diagram. Incomplete core temperature measurement was caused by accidental reasons such as the axillary thermometer falling off.

Of 247 patients screened for eligibility, 243 participants were recruited for the prediction model assessment after excluding 1 participant <18 years old, 2 participants with cervical plexus block, and 1 participant with central hyperthermia. Sixteen participants also were excluded for incomplete perioperative core temperature measurement. Thus, 227 participants were included in the final analysis (Figure 1).

Patients' demographic data are shown in Table 1. The mean ± standard deviation (SD) age was 49 ± 13 years, and the mean BMI was 24.2 ± 3.5 kg/m^2^. The cohort was predominantly women (82.8%) and ASA I (21.1%) or ASA II (69.6%). The two dominant surgery categories were gynecologic surgery (52.4%) and general surgery (26.9%). Most operations were major (35.2%) or major-plus surgery (61.2%). Minimally invasive surgeries (43.6%) were fewer than open surgeries (56.4%). The median (interquartile range [IQR]) duration of anesthesia was 140.5 (108.5–190.0) minutes.

The overall incidence of intraoperative hypothermia was 43.6% (99/227). 18.1% (41/227) of the patients had a temperature of <35.5°C, and 1.3% (3/227) were <35.0°C. The mean ± SD perioperative core temperature decreased from a baseline of 36.56 ± 0.44°C to the lowest of 35.99 ± 0.52°C (Table 2). Among the 227 participants included in the whole cohort, all received intraoperative passive warming, whereas only 10 (4.6%) received intraoperative active warming. The mean ambient temperature in the OR was 22.2 ± 0.9°C. Other outcome data of patient thermometry and warming measures are summarized in Table 2. The overall distribution of the risk score is skewed with a median (IQR) value of 84 (69–91). Risk scores in relation to the incidence of intraoperative hypothermia were demonstrated in Supplemental Digital Content (eFigure 3).

**Figure 2:**
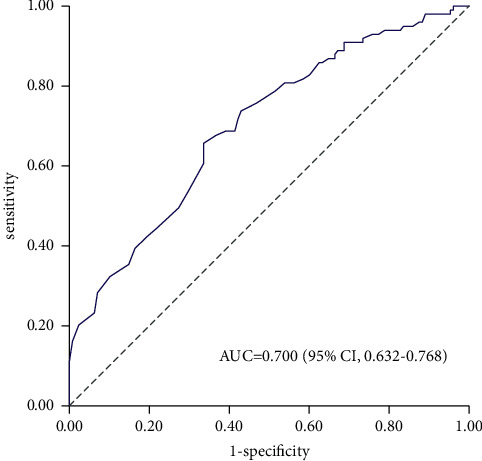
Receiver operating characteristic curve of the prediction model for predicting intraoperative hypothermia in the overall cohort (*N* = 227). An AUC of 0.700 (95% CI, 0.632–0.768) was estimated using the Hanley–McNeil nonparametric method. The gray dashed line represents a model no better than chance (AUC = 0.5). AUC indicates the area under the receiver operating characteristic curve; CI, confidence interval.

The AUC under the ROC curve was 0.700 (95% CI, 0.632–0.768) for all participants (Figure 2), which indicated possible helpful discrimination of the prediction model. Moreover, the model performed well with adequate calibration in terms of the Hosmer–Lemeshow test (*χ*^2^ = 13.8, *P*=0.087) (Supplemental Digital Content, eFigure 4). eFigure 4 also illustrates the calibration plot for 10 pairs of observed and predicted risks. The model tended to overestimate the probability of intraoperative hypothermia in participants with moderate risk, while it underestimated the probability in those with high risk. In addition, a Brier score of 0.33 (95% CI, 0.29–0.37) denoted an acceptable overall performance in all samples.

Table 3 presents the sensitivity, specificity, LR+, LR-, PPV, and NPV at threshold scores of 70 and 90 for the prediction of intraoperative hypothermia in the entire cohort. With participants categorized into 3 risk groups by two cutoffs, the incidence of intraoperative hypothermia was 23.0% (95% CI, 12.4%–33.5%) for the low-risk group, 43.4% (95% CI, 33.7%–53.2%) for the moderate-risk group, and 62.7% (95% CI, 51.5%–74.3%) for the high-risk group; the significant increasing trend was presented across the 3 risk strata (*P* for trend <0.001) (Table 4). According to risk classification, the model could rule out or predict intraoperative hypothermia moderately well (low-risk LR = 0.39 [95% CI, 0.23–0.66]; moderate risk LR = 0.99 [95% CI, 0.74–1.39]; high-risk LR = 2.17 [95% CI, 1.43–3.31]).

In sensitivity analyses for warming status, the model was confirmed to have possibly helpful discrimination (AUC = 0.694; 95% CI, 0.621–0.766) and good calibration (*χ*^2^ = 10.0, *P* = 0.262) after excluding the participants with intraoperative active warming, although the AUC slightly decreased. Analyses that included only temperature data measured by the axillary thermometer revealed that the model had similar discrimination (AUC = 0.704; 95% CI, 0.634–0.774) to that in the whole cohort. However, the model was poorly calibrated (*χ*^2^ = 15.6, *P* = 0.048) among participants whose risk scores were calculated from axillary baseline temperature. The overall performance of the model remained nearly the same in the two subgroups (no intraoperative active warming Brier score = 0.34 [95% CI, 0.29–0.38]; axillary thermometry Brier score = 0.33 [95% CI, 0.29–0.37]).

## 4. Discussion

In this prospective, observational cohort study, we evaluated a previously published prediction model [16] that estimates the risk of intraoperative hypothermia in surgical patients receiving general anesthesia. We found that the model had possibly helpful discrimination, adequate calibration, and acceptable overall performance in the whole data set. Importantly, two practical cutoff scores were determined. The model stratified patients into 3 groups and efficiently identified those at low or high risk of intraoperative hypothermia before the surgery. Given that the prediction model has been modified into an easy-to-use, convenient, and freely accessible mobile APP, it appears, to some extent, ready for further clinical application.

For the function of the model, we emphasize risk stratification rather than estimating the numerical risk for individual patients, so discriminatory power was our main interest rather than calibration [22]. Indeed, a portion of the anesthesiologists knew the preoperative risk assessment value in the study and might be influenced to adjust their routine care of the patients to avoid hypothermia. In addition, in some patients, the use of forced-air warming in proximity to the axillary area might result in falsely elevated axillary temperature that might affect the results. However, these biases tended to weaken and underestimate the accuracy of the prediction, and the model still showed possibly helpful discrimination in our results. Despite sound mean calibration, overprediction or underprediction of the intraoperative hypothermia risk in parts of the patients was also noted through our validation. Thus, the model should not be used to predict the exact probability for a given patient before further refinement with satisfactory calibration at various levels has been conducted [22].

For clinical convenience, 70 and 90 were chosen as cutoff scores in the implementation of the model, yielding the risk classification rules. As demonstrated in our results, the incidence of intraoperative hypothermia in low-risk, moderate-risk, and high-risk groups was 23.0%, 43.4%, and 62.7%, respectively. LRs for the low-risk (0.39) and high-risk groups (2.17) indicated that the model could generate a small but critical change [24] in the pretest probability of intraoperative hypothermia.

Though international standards [1,10] have highlighted the importance of assessing for hypothermia risk in order to plan for perioperative thermal care, few clinical models have attempted to predict perioperative temperature by far [16,26,27], and validation of these rules has been limited [16,26,27]. Clinical guidelines [1,28] have also long established that all surgical patients should be maintaining normothermia via routine temperature measurement and active warming; however, compliance with the guidelines remains poor [11–13], even in developed Western countries, possibly due to unawareness of temperature management [12,13,29] and restricted healthcare resources [12,29]. Besides, Campbell et al. [30] proposed that a ceiling effect may exist when multiple warming methods are used to promote normothermia. Routine forced-air warming may sometimes be redundant, particularly in minor or intermediate surgeries such as cesarean section [14] or endourological procedures [15]. Therefore, the intraoperative hypothermia prediction model might prompt clinicians to keep the high-risk population warm, with comprehensive warming strategies and close monitoring, after preoperative screening [1,10,28], as the risk elevated in our high-risk group to more than 2 times relative to the low-risk group. Meanwhile, the moderate-risk population could be handled with less aggressive thermal care according to the clinical conditions [15]. We believe that the APP has the potential to improve patient outcomes and enhance cost-effective perioperative temperature management, while further clinical impact analysis is required [24].

The mode of patient warming (passive warming vs. active warming) has been reported as a significant predictor of intraoperative hypothermia [12,16,17], but it is not contained in the mobile version of the model. We therefore conducted analyses to assess whether our modified model was sensitive to warming status. Although accuracy is sometimes reasonably lower in sensitivity analyses [31], the model performance was found basically stable in participants who did not develop intraoperative hypothermia. On the other hand, baseline core temperature data might be affected by the differences among various types of thermometers [10,20,28], which could lead to fluctuation in risk scores [16]. The discrimination of the model, limited to participants who had only axillary baseline temperature, remained similar to that in the whole cohort, yet the difference between the observed and predicted risk increased. This difference may be because the model was derived from baseline temperature acquired by infrared tympanic thermometers [12,16], so tympanic membrane thermometry is more appropriate for model calculation than axillary thermometry.

A strength of our study is that it is the first formal prospective validation of an intraoperative hypothermia prediction model with a relatively standard method [24,32] in a real clinical scenario. It is also the first study to accurately place patients into low-risk, moderate-risk, and high-risk classes based on their risk scores and make the explicit probability of intraoperative hypothermia for each group, thus making the model more clinically instructive.

Our study has limitations. First, though predictive performance is expected to be worse with a median AUC decrease of 0.05 during external validation [33]; the AUC was substantially reduced to 0.700 in the current analysis, compared to 0.789 in the internal validation and 0.771 in the external validation after derivation of the model equation [16]. As a result, the model would still render some patients unpredictably hypothermic during surgery. Besides, since the PPVs for the two cutoffs (51.2% and 62.7%) were only slightly higher than the overall incidence of intraoperative hypothermia (43.6%), there is a need to further compare the model with clinicians' intuition. Second, since the study was conducted in several designated operating rooms, this narrow, single-centered cohort had a relatively young, thin, healthy (91% either ASA I or II) set of patients, mainly females, with an operative time of fewer than 2 hours, receiving only general anesthesia, which presents a risk of spectrum bias. Hence, the model could be used only with caution in clinical settings similar to ours after this validation [24]. For greater generalizability, our ongoing multicenter validation study (ClinicalTrials.gov ID: NCT05333120) is prospectively applying this model to a more heterogeneous cohort with patients receiving general anesthesia only or general anesthesia combined with regional anesthesia. Third, in our risk assessment, some of the risk scores were calculated by an investigator but not by clinicians, thus, the feasibility of the model in practice needs to be confirmed in future work, where clinicians apply the model to all patients [24]. Finally, due to a limited number of patients, the model has not been validated in the subgroup that had intraoperative active warming; such warming likely affects the development of intraoperative hypothermia, which could result in a change in discrimination.

## 5. Conclusions

The intraoperative hypothermia prediction model performed relatively well with possibly helpful discrimination and adequate calibration in this prospective validation. This simple and practical model could potentially identify patients at different risks for intraoperative hypothermia, which might help surgical teams to decide clinical protocols for perioperative temperature management.

## Figures and Tables

**Table 1:** Participants' baseline demographics (*N* = 227).

Variable	Value
Patient characteristics	
Age (years)	49 ± 13
Sex, male	39 (17.2%)
Height (cm)	163.5 ± 6.8
Weight (kg)	65.0 ± 11.0
BMI (kg/m^2^)	24.2 ± 3.5

ASA physical status	
1	48 (21.1%)
2	158 (69.6%)
3	21 (9.3%)
4	0 (0.0%)

**Anesthesia/surgery details**	
Type of surgery	
General surgery	61 (26.9%)
Thoracic surgery	17 (7.5%)
Orthopedics surgery	7 (3.1%)
Urology	16 (7.0%)
Neurosurgery	1 (0.4%)
Gynecology	119 (52.4%)
ENT surgery	5 (2.2%)
Ophthalmology	1 (0.4%)

Magnitude of surgery^a^	
Minor	0 (0.0%)
Intermediate	8 (3.5%)
Major	80 (35.2%)
Major-plus	139 (61.2%)

Invasiveness of surgery	
Minimally invasive surgery^b^	99 (43.6%)
Open surgery	128 (56.4%)

Mode of anesthesia	
General anesthesia	227 (100.0%)
Duration of anesthesia^c^ (min, *N* = 220)	140.5 (108.5–190.0)
Duration of surgery^d^ (min, *N* = 220)	107.0 (68.0–149.8)
Blood loss (mL)	50 (0–100)

Data are presented as n/N (% of nonmissing data), mean ± SD, or median (IQR). ASA, American Society of Anesthesiologists; BMI, body mass index; ENT, ear, nose, and throat; IQR, interquartile range; SD, standard deviation. a Magnitude of surgery is categorized as minor surgery (i.e., superficial surgery); intermediate surgery (e.g., excision of varicose vein of leg, laparoscopy, and tonsillectomy); major surgery (e.g., total hysterectomy, laparoscopic cholecystectomy, thyroidectomy, and segmental hepatectomy); and major-plus surgery (e.g., total knee arthroplasty, lung operation, colonic resection, neurosurgery, and cardiac surgery). b Minimally invasive surgery includes laparoscopic surgery, video-assisted thoracic surgery, and others. c Duration of anesthesia is the time from induction to discontinuation of anesthetics. d Duration of surgery is the time from incision to closure.

**Table 2:** Descriptive statistics of perioperative temperature and patient warming (*N* = 227).

Variable	Value
Baseline core temperature prior to anesthesia (°C)	36.56 ± 0.44

Type of thermometry at baseline	
Axillary thermometer	210 (92.5%)
Other^a^	17 (7.5%)
Perioperative lowest temperature (°C)	35.99 ± 0.52
Intraoperative passive warming^b^	227 (100.0%)
Intraoperative active warming^c^ (*N* = 217)	10 (4.6%)
Volume of intraoperative intravenous fluid (mL)	1500 (1100–2200)

Intravenous fluid warming (*N* = 219)	
Unwarmed	144 (65.8%)
Prewarmed	18 (8.2%)
Continuously warmed	57 (26.0%)
Volume of irrigation fluid (mL, *N* = 205)	500 (50–1000)

Irrigation fluid warming (*N* = 151)	
Unwarmed	109 (72.2%)
Prewarmed	42 (27.8%)
Blood transfusion (mL); median (range)	0 (0–1200)

Warming of blood transfusion (*N* = 13)	
Prewarmed	5 (38.5%)
Continuous warming	8 (61.5%)

Ambient temperature (°C)	
Preoperative holding area (*N* = 200)	25.5 ± 2.7
Operating room (*N* = 217)	22.2 ± 0.9
Postanesthesia care unit (*N* = 196)	24.1 ± 1.9

Data are shown as n/N (% of nonmissing data), mean ± SD, or median (IQR), unless otherwise specified. IQR, interquartile range; SD, standard deviation. a Others include an infrared tympanic thermometer and nasopharyngeal probe. b Passive warming includes cotton blankets, surgical drapes, and others. c Intraoperative active warming includes forced-air warming, electric blankets, and others.

**Table 3:** Accuracy of risk score cutoffs for predicting intraoperative hypothermia in all 227 participants undergoing general anesthesia.

Variable (95% CI)	Risk score cutoff	
	70	90
Sensitivity (%)	85.9 (79.0–92.7)	42.4 (32.7–52.2)
Specificity (%)	36.7 (28.4–45.1)	80.5 (73.6–87.3)
LR+	1.36 (1.16–1.58)	2.17 (1.43–3.31)
LR-	0.39 (0.23–0.66)	0.72 (0.59–0.86)
PPV (%)	51.2 (43.6–58.8)	62.7 (51.1–74.3)
NPV (%)	77.0 (66.5–87.6)	64.4 (57.0–71.8)

CI, confidence interval; LR-, negative likelihood ratio; LR+, positive likelihood ratio; NPV, negative predictive value; PPV, positive predictive value.

**Table 4:** Incidence of intraoperative hypothermia across the 3 risk groups in the entire sample (*N* = 227).

Risk group^a^	Observed incidence (%)	Relative risk	*P* value for trend^b^	Likelihood ratio
Low	23.0 (12.4–33.5)	(Reference)	<0.001	0.39 (0.23–0.66)
Moderate	43.4 (33.7–53.2)	1.89 (1.13–3.16)		0.99 (0.74–1.39)
High	62.7 (51.5–74.3)	2.73 (1.66–4.48)		2.17 (1.43–3.31)

Data are shown with (a 95% confidence interval). ^a^Risk group was assessed according to the scale: high, ≥ 90 in score; moderate, 70∼90 in score; low, ≤ 70 in score. ^b^*P* value for trend from logistic regression.

## Data Availability

The datasets generated and/or analyzed during the current study are not publicly available but are available from the corresponding author on reasonable request.

## Supplementary Materials

Supplementary Materials The supplementary materials file contains supplementary figures cited in our manuscript and STARD 2015 checklist.

## References

[1] National Collaborating Centre for Nursing and Supportive Care (UK) (2008). *The Management of Inadvertent Perioperative Hypothermia in Adults*.

[2] Burns S. M., Piotrowski K., Caraffa G., Wojnakowski M. (2010). Incidence of postoperative hypothermia and the relationship to clinical variables. *Journal of PeriAnesthesia Nursing*.

[3] Moola S., Lockwood C. (2011). Effectiveness of strategies for the management and/or prevention of hypothermia within the adult perioperative environment. *International Journal of Evidence-Based Healthcare*.

[4] Kurz A., Sessler D. I., Lenhardt R. (1996). Perioperative normothermia to reduce the incidence of surgical-wound infection and shorten hospitalization. *New England Journal of Medicine*.

[5] Billeter A. T., Hohmann S. F., Druen D., Cannon R., Polk H. C. (2014). Unintentional perioperative hypothermia is associated with severe complications and high mortality in elective operations. *Surgery*.

[6] Frank S. M., Fleisher L. A., Breslow M. J. (1997). Perioperative maintenance of normothermia reduces the incidence of morbid cardiac events. A randomized clinical trial. *JAMA*.

[7] Sun Z., Honar H., Sessler D. I. (2015). Intraoperative core temperature patterns, transfusion requirement, and hospital duration in patients warmed with forced air. *Anesthesiology*.

[8] Leslie K., Sessler D. I., Bjorksten A. R., Moayeri A. (1996). Mild hypothermia alters propofol pharmacokinetics and increases the duration of action of atracurium. *Survey of Anesthesiology*.

[9] Mahoney C. B., Odom J. (1999). Maintaining intraoperative normothermia: a meta-analysis of outcomes with costs. *American Association of Nurse Anesthetists Journal*.

[10] Hooper V. D., Chard R., Clifford T. (2010). ASPAN’s evidence-based clinical practice guideline for the promotion of perioperative normothermia: second edition. *Journal of PeriAnesthesia Nursing*.

[11] Epstein R. H., Dexter F., Hofer I. S. (2018). Perioperative temperature measurement considerations relevant to reporting requirements for national quality programs using data from anesthesia information management systems. *Anesthesia & Analgesia*.

[12] Yi J., Lei Y., Xu S. (2017). Intraoperative hypothermia and its clinical outcomes in patients undergoing general anesthesia: national study in China. *PLoS One*.

[13] Sari S., Aksoy S. M., But A. (2021). The incidence of inadvertent perioperative hypothermia in patients undergoing general anesthesia and an examination of risk factors. *International Journal of Clinical Practice*.

[14] Chebbout R., Newton R. S., Walters M., Wrench I., Woolnough M. (2017). Does the addition of active body warming to in-line intravenous fluid warming prevent maternal hypothermia during elective caesarean section? A randomised controlled trial. *International Journal of Obstetric Anesthesia*.

[15] Azhar F., Dyer J. E., Clarke L. (2019). Is it necessary to use forced air warming in core endourological procedures where warmed intravenous and irrigation fluids are routinely used. *Journal of Clinical Urology*.

[16] Yi J., Zhan L., Lei Y. (2017). Establishment and validation of a prediction equation to estimate risk of intraoperative hypothermia in patients receiving general anesthesia. *Scientific Reports*.

[17] Yi J., Xiang Z., Deng X. (2015). Incidence of inadvertent intraoperative hypothermia and its risk factors in patients undergoing general anesthesia in Beijing: a prospective regional survey. *PLoS One*.

[18] Bossuyt P. M., Reitsma J. B., Bruns D. E. (2015). STARD 2015: an updated list of essential items for reporting diagnostic accuracy studies. *BMJ*.

[19] Pei L., Huang Y., Mao G., Sessler D. I. (2018). Axillary temperature, as recorded by the iThermonitor WT701, well represents core temperature in adults having noncardiac surgery. *Anesthesia & Analgesia*.

[20] Sessler D. I. (2021). Perioperative temperature monitoring. *Anesthesiology*.

[21] Haukoos J. S., Newgard C. D. (2007). Advanced statistics: missing data in clinical research--part 1: an introduction and conceptual framework. *Academic Emergency Medicine*.

[22] Alba A. C., Agoritsas T., Walsh M. (2017). Discrimination and calibration of clinical prediction models: users’ guides to the medical literature. *JAMA*.

[23] Hanley J. A., McNeil B. J. (1982). The meaning and use of the area under a receiver operating characteristic (ROC) curve. *Radiology*.

[24] Guyatt G., Rennie D., Meade M. O., Cook D. J. (2015). *Users’ Guides to the Medical Literature: A Manual for Evidence-Based Clinical Practice*.

[25] Steyerberg E. W., Vickers A. J., Cook N. R. (2010). Assessing the performance of prediction models: a framework for traditional and novel measures. *Epidemiology*.

[26] Mendonça F. T., Ferreira J. D. S., Guilardi V. H. F., Guimaraes G. M. N. (2021). Prevalence of inadvertent perioperative hypothermia and associated factors: a cross-sectional study. *Therapeutic Hypothermia and Temperature Management*.

[27] Severens N. M. W., Lichtenbelt W. D. v M., Frijns A. J. H., Steenhoven A. A. V., Mol B. A. J., Sessler D. I. (2007). A model to predict patient temperature during cardiac surgery. *Physics in Medicine and Biology*.

[28] Forbes S. S., Eskicioglu C., Nathens A. B. (2009). Evidence-based guidelines for prevention of perioperative hypothermia. *Journal of the American College of Surgeons*.

[29] Munday J., Delaforce A., Forbes G., Keogh S. (2019). Barriers and enablers to the implementation of perioperative hypothermia prevention practices from the perspectives of the multidisciplinary team: a qualitative study using the Theoretical Domains Framework. *Journal of Multidisciplinary Healthcare*.

[30] Campbell G., Alderson P., Smith A. F., Warttig S. (2015). Warming of intravenous and irrigation fluids for preventing inadvertent perioperative hypothermia. *Cochrane Database of Systematic Reviews*.

[31] Saltelli A., Scott M. (1997). Guest editorial: the role of sensitivity analysis in the corroboration of models and its link to model structural and parametric uncertainty. *Reliability Engineering & System Safety*.

[32] Laupacis A., Sekar N., Stiell I. G. (1997). Clinical prediction rules. A review and suggested modifications of methodological standards. *JAMA, the Journal of the American Medical Association*.

[33] Siontis G. C., Tzoulaki I., Castaldi P. J., Ioannidis J. P. (2015). External validation of new risk prediction models is infrequent and reveals worse prognostic discrimination. *Journal of Clinical Epidemiology*.

